# Angiogenin and vascular endothelial growth factor expression in lungs of lung cancer patients

**DOI:** 10.2478/v10019-012-0031-1

**Published:** 2012-11-09

**Authors:** Ales Rozman, Mira Silar, Mitja Kosnik

**Affiliations:** 1 Department for Endoscopy; 2 Department for Immunology; 3 Department for Pulmonology, University Clinic Golnik, Slovenia

**Keywords:** angiogenin, BAL fluid, bronchoscopy, lung cancer, vascular endothelial growth factor

## Abstract

**Background.:**

Lung cancer is the leading cause of cancer deaths. Angiogenesis is crucial process in cancer growth and progression. This prospective study evaluated expression of two central regulatory molecules: angiogenin and vascular endothelial growth factor (VEGF) in patients with lung cancer.

**Patients and methods.:**

Clinical data, blood samples and broncho-alveolar lavage (BAL) from 23 patients with primary lung carcinoma were collected. BAL fluid was taken from part of the lung with malignancy, and from corresponding healthy side of the lung. VEGF and angiogenin concentrations were analysed by an enzyme-linked immunosorbent assay. Dilution of bronchial secretions in the BAL fluid was calculated from urea concentration ratio between serum and BAL fluid.

**Results.:**

We found no statistical correlation between angiogenin concentrations in serum and in bronchial secretions from both parts of the lung. VEGF concentrations were greater in bronchial secretions in the affected side of the lung than on healthy side. Both concentrations were greater than serum VEGF concentration. VEGF concentration in serum was in positive correlation with tumour size (p = 0,003) and with metastatic stage of disease (p = 0,041). There was correlation between VEGF and angiogenin concentrations in bronchial secretions from healthy side of the lung and between VEGF and angiogenin concentrations in bronchial secretions from part of the lung with malignancy.

**Conclusion.:**

Angiogenin and VEGF concentrations in systemic, background and local samples of patients with lung cancer are affected by different mechanisms. Pro-angiogenic activity of lung cancer has an important influence on the levels of angiogenin and VEGF.

## Introduction

Lung cancer is one of the leading causes of morbidity and cancer-related mortality in Slovenia with 1216 new cases and 1125 deaths in 2007.^1^ Five-year survival rate is around 10%, since most of the patients have advanced stage of disease at the time of diagnosis.^2^,^3^ Tumour growth and progression is still poorly understood and in many cases doesn’t follow currently accepted model of sequential multistep pathway.^4^–^7^

Beside transformation of normal cells into malignant, the cancer growth and progression requires additional steps to transition from dormant to malignant state.^8^,^9^ The role of angiogenesis – the growth of new vessels from preexisting – is probably fundamental in this process.^10^,^11^ Angiogenesis is controlled by a delicate balance between pro- and anti-angiogenic factors which is disrupted by a malignant tumour in favour of forming its own blood vessel network.^12^,^13^ Moreover, angiogenesis is involved also in the growth of distant metastases.^14^

The central roles in the process of neovascularisation have regulatory molecules with pro- and anti-angiogenic potential.^15^ Vascular endothelial growth factor (VEGF) and angiogenin are two major positive regulators of blood vessel formation in physiological conditions and in pathological situations including lung cancer.^16^–^20^

The purpose of this study was to prospectively investigate the local concentrations of VEGF and angiogenin in the part of the lung, affected by a lung cancer and compare them by the healthy side of the lung and by the concentrations in the serum of patients with lung cancer.

## Patients and methods

### Patients

Thirty patients who underwent bronchoscopy with suspicion for lung malignancy were included. Exclusion criteria were: age under 18 years, malignancy on both sides of the lung or additional malignoma elsewhere in the body, previous anticancer treatment or pneumonectomy. The procedure was explained to patients verbally and in writing, and signed informed consent was obtained prior to enrolment. The study was approved by the National Medical Ethics Committee. We subsequently excluded 7 patients where lung malignancy was not confirmed by invasive diagnostic procedures. Clinical and demographic characteristics of the included lung cancer patients are presented in Table 1. Ex-smokers were defined as former regular smokers, who stopped at least one year before enrolment.

### Broncho-alveolar lavage (BAL) and blood samples

Venous blood samples were collected before bronchoscopy into a vacutainer without anticoagulant or other additives. Serum was separated and centrifuged at 2500 revolutions per minute for 5 minutes, and supernatant was frozen at − 40°C. During bronchoscopy BAL with 20 ml of saline was collected from segmental bronchus affected by lung cancer and from corresponding segmental bronchus on the healthy side of the lung. BAL was performed before biopsies, from the healthy side first to avoid contamination with blood and malignant cells. BAL fluid was centrifuged at 1600 revolutions per minute for 5 minutes after procedure and supernatant was frozen at − 40°C for later analysis.

### VEGF, angiogenin and urea measurement

Enzyme-linked immunosorbent assay (ELISA) kits (Quantikine – R&D Systems, Minneapolis) were used for measurements of VEGF and angiogenin in plasma and BAL fluid. The limit of sensitivity of the VEGF assay was 5–9 pg/ml, the intra-assay coefficient of variation was 4.1–6.7% and the interassay coefficient of variation was 5.0–8.8%. The limit of sensitivity of the angiogenin assay was 6 pg/ml, the intra-assay coefficient of variation was 2.8–3.3% and the interassay coefficient of variation was 7.1 – 8.7%. Urea concentration in plasma and BAL fluid was measured by QuantiChrom urea assay kit Diur-500 (BioAssay Systems, Hayward) with linear detection range from 0.06 mg/l to 1000 mg/l.

### Data analysis

Concentrations of angiogenin and VEGF in bronchial samples were normalized to urea concentration. Concentrations of VEGF and angiogenin in bronchial secretions were calculated using formula: bronchial secretions concentration = BAL fluid concentration x (urea concentration in plasma / urea concentration in BAL fluid).

Data were analysed using the computer program Statistical Package for the Social Sciences version 14.0 (SPSS Inc., Chicago). Descriptive statistical methods were used (mean, standard deviation, range / median, percentiles). A Kolmogorov-Smirnov (KS) test was used to check normal distribution. Associations between variables were tested by Pearson non-parametric test and by Wilcoxon matched-pairs test. Differences were analysed by Mann-Whitney U test. All tests were two-tailed; *p* < 0.05 was considered to be statistical significant.

## Results

Median dilution of bronchial secretions in BAL fluid was 1:104 on the healthy side of the lung and 1:97 in the area of the lung affected by lung cancer, according to differences in urea concentrations.

Only serum concentrations of angiogenin followed Gaussian distribution (KS = 0.16, p > 0.05).

**FIGURE 1:**
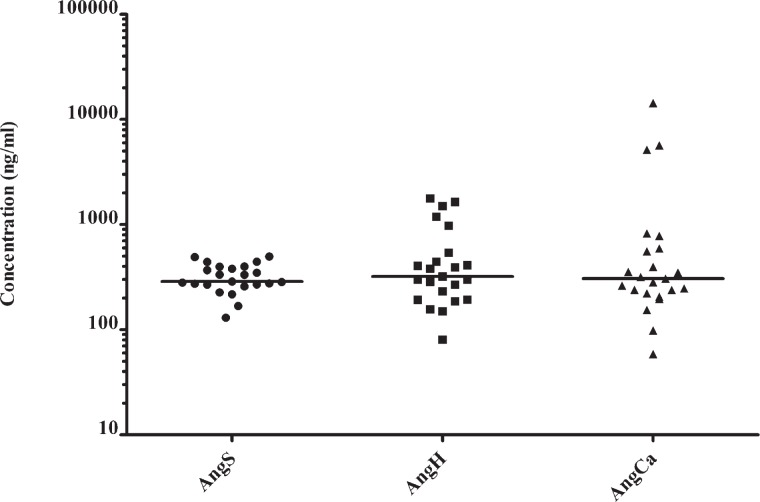
Distributions of angiogenin concentrations in serum (AngS), bronchial secretions from healthy side of the lung (AngH) and from area, affected by lung cancer (AngCa).

Median angiogenin serum concentration in patients with lung cancer was 286.94 ng/ml (Table 2). We found no significant correlation among angiogenin concentrations in serum and bronchial secretions of both sides of the lung (p > 0.05). No significant difference was found among angiogenin concentrations in serum and bronchial secretions of both sides of the lung (p > 0.05) (Figure 1).

**FIGURE 2:**
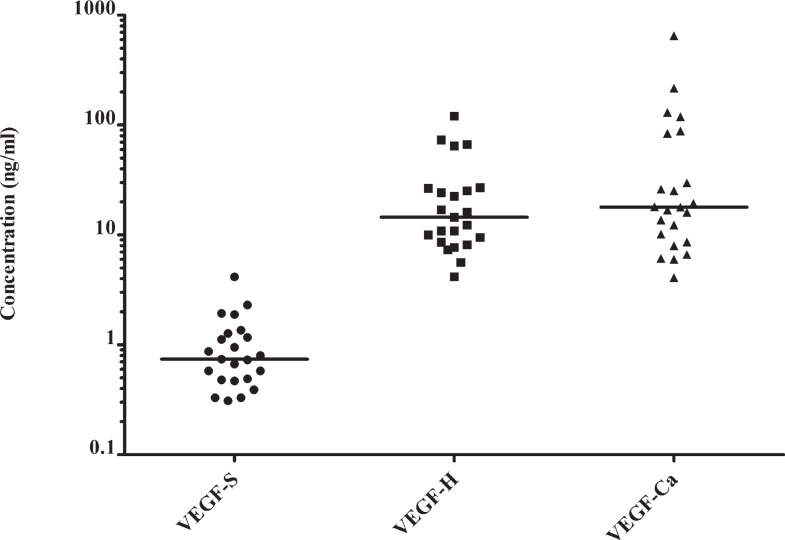
Distributions of VEGF concentrations in serum (VEGF-S), bronchial secretions from healthy side of the lung (VEGF-H) and from area, affected by lung cancer (VEGF-Ca).

VEGF concentrations in bronchial secretions were higher than in the serum of patients with lung cancer (Table 3). VEGF concentrations in the bronchial secretions from lung cancer area show no statistical difference to VEGF concentrations in bronchial secretions from the healthy side of the lung (Figure 2). There was no significant correlation among VEGF concentrations in serum and bronchial secretions found (p > 0.05).

Concentrations of angiogenin and VEGF in bronchial secretions from the healthy side of the lung were in a correlation (p = 0.003) as well as angiogenin and VEGF concentrations from area affected with lung cancer (p < 0.001). Angiogenin and VEGF concentrations in the serum did not correlate.

Concentrations of serum VEGF were in a positive correlation with tumour size (p = 0.003), plasma C-reactive protein (CRP) concentrations (p = 0.007), white blood count (WBC) in peripheral blood sample (p < 0.001) and metastatic stage of disease (p = 0.041). Serum angiogenin concentrations were not in a correlation with any of tumour features or inflammatory markers.

## Discussion

The study showed that there is a strong local production of VEGF in lungs of patients with lung cancer and that VEGF serum concentration but not angiogenin concentration was in positive correlation with tumour size and with metastatic stage of disease. Concentrations of VEGF and angiogenin did not correlate for a particular molecule in any possible couple from three taken samples in patients with lung cancer. This is probably a consequence of different mechanisms, which regulate concentrations of these two molecules in any of these three settings.

Serum as a systemic sample has certain levels of VEGF and angiogenin present also in subjects without malignant disease and their concentration is therefore dependant on mechanisms other than malignancy, such are inflammation, hypoxemia, injury and tissue repair and maintenance.^21^,^22^

Angiogenin is normally present in the serum of healthy people and is produced in many tissues, among other in the liver as a constituent of acute phase response.^23^,^24^ Its concentrations are higher in heart failure, abnormal pregnancy, blood malignancies and in some other conditions.^25^,^26^ Higher expression of angiogenin was found in bronchial mucosa of asthmatics, especially during periods of exacerbation.^27^,^28^ In this study serum angiogenin concentrations followed Gaussian distribution in contrast to angiogenin concentrations in bronchial secretions from both sides of the lung. Therefore we assume that serum angiogenin concentration was not significantly affected by local conditions in the lung and in the lung cancer. Moreover serum angiogenin concentrations were not in correlation with any of the clinical variables as for example: CRP, WBC, tumour size, stage of the disease, chronic obstructive lung disease (COPD), etc.

In contrast, VEGF concentrations in the serum were much lower than in the samples from the lung, and were not in correlation with lung concentrations. Serum VEGF concentrations were strongly correlated with some clinical variables: CRP, WBC, tumour size and stage of the disease. This is in accordance with previously described higher serum VEGF expression in patients with advanced disease.^29^,^30^ Serum concentrations of VEGF are normally very low and are probably the result of “washing” from tissues, where VEGF expression is higher.^31^,^32^

The measurements of exact concentrations of angiogenin and VEGF in bronchial secretions represented a certain challenge, since dilution rate of bronchial secretions in BAL was different from sample to sample. Angiogenin and VEGF concentrations in BAL fluid were dependant on their concentrations in bronchial secretions and on the amount of bronchial secretions captured in the retrieved BAL fluid. For defining of exact dilution rate of secretions in the BAL we used concentrations of urea molecules and two compartment model method. Urea freely diffuses between plasma and bronchial secretions and its concentration in both fluids is equal, since urea is neither degraded or produced in bronchi nor is volatile.^33^–^35^

Bronchial secretions from the healthy side of the lung were regarded as a “lung background” sample. Correlation between angiogenin and VEGF concentrations in bronchial secretions from the healthy side of the lung is probably the consequence of the same stimulus. Bronchial inflammation in asthma and COPD can stimulate secretion of angiogenin and VEGF.^27^,^28^ VEGF concentrations in bronchial secretions can rise even in asymptomatic smokers.^36^ Almost all of the patients in this study were current or former smokers and 60.9% had diagnosis of COPD that could contribute especially to higher VEGF concentrations.

Bronchial secretions from the area of the lung, affected with lung cancer were “local” sample. Concentrations of angiogenin and VEGF were in correlation and we again assume that stimulus for secretion could be the same. VEGF concentrations were higher in bronchial washings from patients with lung cancer after treatment with chemotherapy and irradiation, but none of included patients in this study received such treatment before.^37^ VEGF concentrations in bronchial secretions from affected side of the lung were not in correlation with any of the tumour features, what possibly reflects the effect of VEGF “washing” to the blood serum, which in contrast reflects such correlations. We were unable to confirm the findings that VEGF expression in the airways is inversely associated with tumour progression.^38^

Limitations of the study were small number of included patients, and inhomogenity of the study group. Small sample size prevented subgroup analysis. In the study group were differences in gender and histological types of lung cancer with regard to general distribution in population of patients with lung cancer.

We summarize that angiogenin and VEGF expression in systemic, background and local samples of patients with lung cancer are affected by different mechanisms. The important role in serum angiogenin concentration has constitutional component in contrast to serum VEGF concentration, which is influenced by lung cancer. Angiogenin and VEGF concentrations in bronchial secretions from healthy and affected side of the lung show influence of lung cancer angiogenic activity and other conditions including co-morbidity.

## Figures and Tables

**TABLE 1:** Clinical and demographic characteristics of the lung cancer patients from the study

**Variables**	**Data**
No. of patients	23
Median age, years	66 (range 48–78)
Male, *n* (%)	22 (95.7%)
Female, *n* (%)	1 (4.3%)
Smoking status, *n* (%)	
Current and previous	22 (95.7%)
Never-smoker	1 (4.3%)
COPD, *n* (%)	14 (60.9%)
Cancer type	
Squamous cell carcinoma	13 (56.6%)
Small cell carcinoma	5 (21.7%)
Adenocarcinoma	3 (13.0%)
Others	2 (8.7%)
Stage, *n* (%)	
I	4 (17.4%)
II	1 (4.3%)
III	8 (34.8%)
IV	10 (43.5%)
Tumour size, cm	5.7 ± 3.0
Location, *n* (%)	
central	16 (69.6%)
peripheral	7 (30.4%)
CRP, mg/l	36.5 ± 32.8
WBC, ×10^6^/l	11.1 ± 9.6

COPD = chronic obstructive lung disease; CRP = C-reactive protein; WBC = white blood count

**TABLE 2:** Concentrations of angiogenin in serum and in bronchial secretions from healthy side of the lung and from area, affected by lung cancer

**Angiogenin concentration**	**Me**	**Q1**	**Q3**	**95.centil**
Serum (ng/ml)	286.94	267.88	397.08	495.88
Bronchial secretions – healthy side (ng/ml)	320.64	193.38	538.70	1746.17
Bronchial secretions – lung cancer (ng/ml)	306.11	221.19	592.69	12591.24

Me = median, Q1 = first quartile, Q3 = third quartile

**TABLE 3:** Concentrations of VEGF in serum and in bronchial secretions from healthy side of the lung and from area, affected by lung cancer. There was a significant difference between serum and bronchial concentrations (p < 0.05), but no significant difference among VEGF concentrations between diseased and healthy side of the lung (p > 0.05)

**VEGF concentration**	**Me**	**Q1**	**Q3**	**95.centil**
Serum (ng/ml)	0.74	0.48	1.27	3.79
Bronchial secretions – healthy side (ng/ml)	14.52	8.59	26.59	111.05
Bronchial secretions – lung cancer (ng/ml)	17.89	8.64	84.30	565.49

Me – median, Q1 – first quartile, Q3 – third quartile
